# Association between Auricular Signals and the Risk Factors of Metabolic Syndrome

**DOI:** 10.3390/medicines4030045

**Published:** 2017-06-25

**Authors:** Lorna Kwai Ping Suen, Chao Hsing Yeh, Simon Kai Wang Yeung, Jojo Yee Mei Kwan, Hon Fat Wong, David Chan, Alice Siu Ping Cheung, Vincent Tok Fai Yeung

**Affiliations:** 1 School of Nursing, Hong Kong Polytechnic University, Hong Kong, China; simonyeung.kw@gmail.com (S.K.W.Y.); hon.fat.dino.wong@polyu.edu.hk (H.F.W.); seeninepwh@yahoo.com (A.S.P.C.); 2 Associate Professor, Acute and Chronic Care, Johns Hopkins School of Nursing, Baltimore, MD 21205, USA; cyeh13@jhu.edu; 3 Centre for Diabetes Education & Management, Our Lady of Maryknoll Hospital, Hong Kong, China; kwanym3@ha.org.hk; 4 Kinetics Medical & Health Group Co. Ltd., Hong Kong, China; davidchan@kinetics.hk; 5 Department of Medicine & Geriatrics, Our Lady of Maryknoll Hospital, Hong Kong, China; yeungtf@ha.org.hk

**Keywords:** Auricular diagnosis, metabolic syndrome, auricular signals, screening, diabetes, cardiovascular

## Abstract

**Objective:** This study aims to determine the association between auricular signals and the risk factors of metabolic syndrome (MS). **Methods:** A case-control study with an equal number of cases and controls matched by age group and gender was conducted. A total of 204 participants were recruited. Patients were verified as having MS based on the International Diabetes Federation (IDF) criteria. Auricular assessment was conducted in the following sequence: visual inspection, electrical skin resistance test (ESRT), and pressure pain test (PPT). **Results:** MS+ patients tend to have much more oily auricle complexion than the controls. The ‘endocrine’ (right) of the participants with MS indicated a significantly higher electrical conductivity compared to that of the controls. The MS group participants experienced significant tenderness on the ‘heart’ and ‘endocrine’ acupoints. A number of auricular signals were also associated with the risk factors of MS, including age, gender, smoking status, family history of diabetes, and comorbid illnesses. Both the ‘heart’ and ‘endocrine’ acupoints showed the highest sensitivity to tenderness (60.8%), followed by the ‘endocrine’ (59.8%) and ‘pancreas and gallbladder’ (55.9%). **Conclusions**: The results of this study suggest that electrical conductivity and tenderness of a number of auricular points, including the ‘heart’, ‘pancreas and gall bladder’, and ‘endocrine’, are associated with MS and its risk factors. Further investigations with a larger sample size could be conducted to verify the value of these auricular signals on MS risk prediction so that this method can be used as an early screening method for the population with a high MS risk.

## 1. Introduction

Metabolic syndrome (MS) is a cluster of multiple abnormalities that are associated with insulin resistance, central obesity, high blood pressure, and dyslipidaemia (i.e., hypertriglyceridaemia and low high-density lipoprotein cholesterol (HDL-C)) [1,2]. Many individuals do not realize that they manifest one or more of the MS indicators until they undergo blood screening and physical assessment. MS is possibly associated with the onset of diabetes and cardiovascular disease, which are common chronic illnesses worldwide. The probability of having cardiovascular diseases increases up to 31.2% among people having two MS abnormalities and up to over 40% among those with four or five MS abnormalities [3].

Early identification of MS status could markedly reduce possible negative health consequences and related complications. Therefore, early screening with a fast, non-invasive, and cost-effective approach is necessary. Previous researchers have proposed the use of simplified methods, such as the homeostasis model assessment (HOMA), which was originally designed to assess insulin resistance. However, the HOMA index is an invasive method that requires blood testing [4]. Recently, the ^13^C-glucose breath test (C-GBT) has been proposed as a valid non-invasive screening tool to identify MS in adolescents [5]. This method is based on the assumption that subjects with impaired glucose metabolism or type II diabetes mellitus (T2DM) will have a lesser CO_2_ amount in their breath owing to an impaired glucose uptake. However, maldigestion of specific carbohydrates, such as lactose and fructose, may lead to a false-positive result [6].

Auricular diagnosis is an objective, painless, and non-invasive screening method. The reflexive property of the ear can cause various physical attributes to appear on the auricle in the presence of bodily disorders. Such attributes include variations in shape, colour, size, and sensation; formation of papules, creases, and oedema; and increased tenderness or decreased electrical conductivity [7,8]. In the auricular diagnosis system, the areas of the auricle with increased electrical conductivity and tenderness when touched correspond to specific areas of the body where some pathological conditions are present [8,9]. The areas where electrical resistance is lower than the normal level are considered as either positively or highly conductive electrical points [9,10]. 

Auricular signs associated with body functions have been described in previous studies. Abnormal hair growth in the ear is a sign of hormonal changes associated with the decrease in the energy flow of the kidney, which occurs with age [7]. The presence of an ear lobe crease and ear hairs is significantly related to coronary heart disease [9,11]. In a case–control study conducted by Suen et al. [12], the tenderness and electrical conductivity of some auricular points, including the ‘pancreas and gallbladder’, ‘endocrine’, ‘kidney’, ‘lower tragus’, ‘heart’, and ‘eyes’, were linked to T2DM in the Chinese population. Considering that auricular diagnosis is an objective, painless, and non-invasive method that has a pre-diagnostic value to facilitate screening, the association between auricular signals and MS was further investigated. This study aims to determine the association between auricular signals and the risk factors of MS. The hypotheses of this study are as follows: (1) visual inspection results indicate that participants with MS+ exhibit a higher occurrence of ear lobe crease, hair growth, and oily ear complexion compared with those who are MS−; (2) participants with MS+ have lower electrical skin resistance on specific auricular points (‘heart’, ‘pancreas and gallbladder’, and ‘endocrine’) of the ears than those who are MS−; (3) participants with MS+ have significant tenderness on specific auricular points (‘heart’, ‘pancreas and gallbladder’, and ‘endocrine’) of the ears than MS− participants.

## 2. Materials and Methods 

### 2.1. Settings and Participants

A total of 204 participants were recruited in this matched case–control study. This group comprised of 102 MS cases and an equal number of controls who are matched by age group and gender. Patients were verified as having MS based on the International Diabetes Federation (IDF) criteria, which include the presence of central obesity (i.e., waist circumference ≥90 cm (35 inches) in Asian men or ≥80 cm (31 inches) in Asian women (if body mass index (BMI) is >30 kg/m^2^; central obesity could be assumed without the need to refer to waist circumferences) and at least two of the following criteria: (1) triglyceride concentration ≥150 mg/dL (1.7 mmol/L) or specific treatment for lipid abnormality; (2) high-density lipoprotein ≤40 mg/dL (1.03 mmol/L) in men or ≤50 mg/dL (1.29 mmol/L) in women or specific treatment for lipid abnormality; (3) systolic blood pressure ≥130 mmHg or diastolic blood pressure ≥85 mmHg or treatment of previously diagnosed hypertension; and (4) fasting plasma glucose ≥100 mg/dL (5.6 mmol/L) or previously diagnosed T2DM [13]. Controls were defined as individuals who do not manifest MS based on the aforementioned criteria. The age distributions taking into consideration the accessibility of participants in the venues were as follows: 40 to 49 (*n* = 20, 10%), 50 to 59 (*n* = 72, 35%), 60 to 69 (*n* = 72, 35%) and 70 or above (*n* = 40, 20%). Participants with aural injury, deformities, or infections that might affect the ear appearance and shape were excluded from the study.

Participants were recruited through convenience sampling from the Centre for Diabetes Education and Management of a regional hospital in Hong Kong (*n* = 108), an integrated health clinic of a university (*n* = 85) and a private laboratory (*n* = 11). The patients’ medical histories that covered the common risk factors of MS, including age, gender, BMI, systolic and diastolic blood pressure, family history, and medical conditions were collected. Laboratory examinations on fasting plasma glucose, triglyceride concentration, and lipid profile were taken. 

### 2.2. Selection of Specific Auricular Points

Points were selected owing to their possible association with MS pathophysiology. The ‘heart’ was chosen because this organ is the acupoint associated with cardiovascular conditions, such as hypertension or coronary heart disease [11]. The ‘pancreas and gallbladder’ is the acupoint related to hyperglycaemia caused by insulin resistance [14], whereas hyperlipidaemia is one of the risk factors of MS and is commonly associated with endocrine disorders, such as obesity and genetic or metabolic conditions [15]. The presence of an ear lobe crease and ear hairs has been reported to have significant association with coronary heart disease [9,11]. In addition, people with metabolic disorders may tend to have oily complexion (verbal communication with one of the co-authors, HFW, who is a registered traditional Chinese medicine (TCM) practitioner). Therefore, these auricular signals were evaluated.

**Figure 1:**
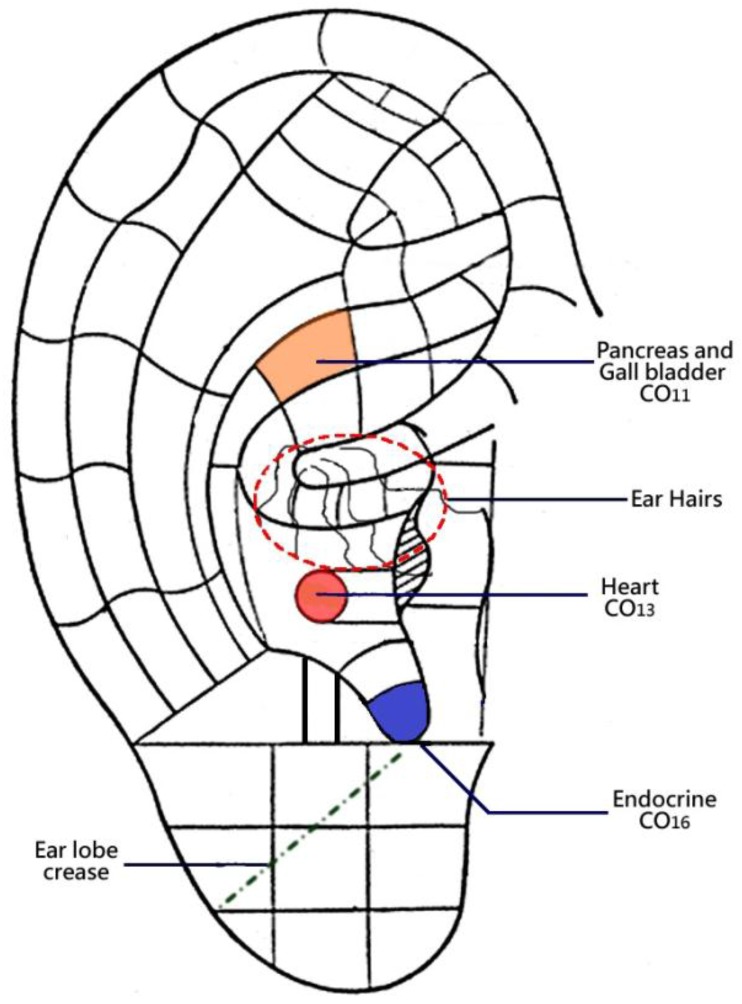
Auricular points and signals for testing the association of metabolic syndrome.

The Chinese Standard Ear-Acupoints Chart GB/T13734-2008) [16], which is recognised by the World Health Organization, is used for acupoint location. Figure 1 displays the location of these specific auricular points in the Chinese Standard Ear-Acupoint Chart.

### 2.3. Auricular Assessment

Auricular assessment was conducted in the following sequence: visual inspection, electrical skin resistance test (ESRT), and pressure pain test (PPT). The researcher, who is a registered TCM practitioner (HFW), involved in the auricular assessment was blinded to the grouping of the subjects.

a) Visual Inspection

Both auricles were observed for the presence or absence of oily complexion, ear hairs, and ear lobe creases. An ear lobe crease is tagged as present if a visible wrinkle, either unilaterally or bilaterally, runs from the lower probe of the external auditory meatus diagonally backward to the edge of the lobe at approximately 45 degrees [17].

b) ESRT

Before conducting ESRT, an individual threshold using the ‘shenmen’ ear point as a reference was used. ‘Shenmen’ was chosen as the reference point because it is usually reactive to the effects of everyday stress in a person’s life and is consistently identified in most people [18]. The procedure involved setting the threshold by placing the acupoint detector ‘Pointer Plus’ [18] on the ‘shenmen’ and increasing the detection sensitivity until the equipment indicated high electrical conductance. The sensitivity was then slightly reduced until the ‘shenmen’ was only barely picked up [10,19]. After the identification of the ‘shenmen’ point sensitivity, the electrical skin resistance of the three specific acupoints (‘heart’, ‘pancreas and gallbladder’, and ‘endocrine’) were measured. The result was taken as ‘positive’ when the acupoints under testing have a higher conductivity compared with the ‘shenmen’ (reference point), and vice versa.

c) PPT

A pressure algometer with a unit range of 0–500 g was used to apply force on the ‘shenmen’ point, which was taken as a reference to compare the tenderness experienced by the participants on the specific points (‘heart’, ‘pancreas and gallbladder’, and ‘endocrine’). The researcher recorded the observed value (g) when the subject started to feel a tender sensation upon the application of the instrument’s pointer on the acupoints under testing. The result was ‘positive’ when the acupoints under testing displayed a lower tolerance to tenderness compared with the ‘shenmen’ (reference point), and vice versa.

### 2.4. Validity and Reliability

During the testing, the examination techniques were also observed. For example, moving the detector too quickly can easily miss a reactive auricular point, whereas applying too much pressure with the detector may create false ear points merely because of the increased contact with the skin. The detector should be held perpendicular to the stretched surface of the ear and gently gliding it over the ear during examination. No ear cleaning should be done before testing. If the examination was conducted in cold weather, the person receiving the examination should rest for at least 10 min in room temperature before the procedure [12]. The inter-observer agreement between two researchers (LS and HFW) was recorded for 10 cases to assess the reliability of the auricular examinations, and a 92% agreement was achieved. If discrepancies in the assessment occurred between the two raters, a consensus was sought after discussion. The photos of the auricles were also taken using a digital camera to facilitate the cross-checking of ratings on the visual inspection of the auricles on other participants. During the auricular examination, the researchers conducting the auricular examination were blinded to the subject grouping to avoid observer bias.

### 2.5. Data Analyses

Descriptive statistics was used to present the sociodemographic characteristics, family history, and medical conditions of the participants. McNemar’s test was utilised to determine the auricular signal association between cases and controls. Chi-square test was performed to examine the association between auricular signals and risk factors of MS. The sensitivity, specificity, and positive and negative predictive values of the auricular signals on the risk of MS were also computed. Analyses were conducted using SPSS Statistics 23. A *p* < 0.05 was considered statistically significant.

### 2.6. Ethical Considerations

Ethical approval from the study venues and the university was sought. Written informed consent was obtained from all participants. The purpose and procedures of the study were explained verbally and in writing to the participants. Participation in the study was voluntary, and all participants were assured that they have the right to refuse or withdraw from the study at any time. Personal information and data remained confidential and anonymous.

## 3. Results

### 3.1. Demographic Characteristics, Family History, and Medical Conditions of the Participants

A total of 204 Chinese participants were evaluated, with an equal number of cases (*n* = 102) matched with controls (*n* = 102) based on age group and gender. The mean age was 61.16 years (SD = 10.33). No significant differences were observed in the employment status, smoking status, and alcohol consumption of all the participants. The mean BMI of the participants in the MS and control groups were 27.06 (SD = 3.87) and 22.85 (SD = 2.64), respectively. Statistically significant differences between groups were noted in terms of the presence of central obesity, family history on diabetes/hyperlipidaemia/hypertension, and diabetes/coronary heart disease/hypertension and the occurrence of comorbid illness (Table 1).

### 3.2. Association of Auricular Signals between Cases and Controls

**Figure 2:**
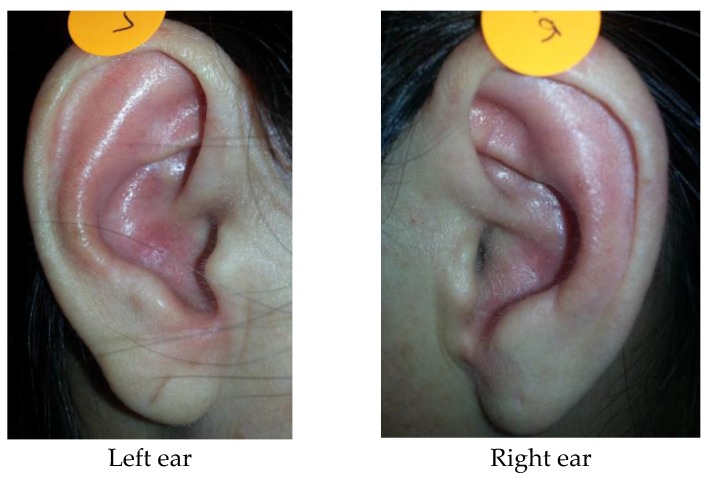
Oily complexion of the auricles in a participant with metabolic syndrome (right and left ears).

The auricular signals observed in the participants of the MS+ group were compared with those of the participants in the control group (MS−) using three auricular examination methods (Table 2):

a) Visual inspection: MS+ participants tend to have more oily auricle complexion, on either the right (*p* < 0.001) or left side of the ears (*p* < 0.001), than the controls (Figure 2). However, no significant association was observed in the ear hairs and ear lobe creases between the groups.

b) ESRT: among the three acupoints under testing, only the ‘endocrine’ (right) acupoint of the participants with MS indicated a significantly higher conductivity compared to that of the participants in the control group.

c) PPT: the MS group participants experienced significant tenderness on the ‘heart’ (right and left sides) and ‘endocrine’ acupoints (right and left).

### 3.3. Association between Auricular Signals and Risk Factors of MS

A number of auricular signals were associated with the risk factors of MS. For example, the oily complexion of ears was related to coronary heart disease and family history of diabetes and central obesity. In addition, the electrical conductivity of most acupoints under testing were significantly associated with the risk factors of MS, including age, gender, smoking status, family history of diabetes, and comorbid illnesses (diabetes/hypertension/coronary heart disease (CHD)/hyperlipidaemia). The tenderness of ‘pancreas and gallbladder’ and ‘endocrine’ was also linked to a number of MS risk factors (Table 3).

### 3.4. Association between Auricular Signals under Testing

Significant associations between auricular signals under testing were identified. For example, the presence of ear hairs or ear lobe crease was associated with the electrical conductivity of many acupoints under testing. It was also observed that the acupoints on the left or right ears were highly associated in terms of the electrical conductivity detected by the ‘Pointer plus’ or tenderness perceived by the participants (Table 4).

### 3.5. Predictive Power of Auricular Signals on the Risk of MS

Among the auricular signals under testing, both the ‘heart’ (right) and ‘endocrine’ (right) showed the highest sensitivity to tenderness (60.8%), followed by the ‘endocrine’ (left) (59.8%) and ‘pancreas and gallbladder’ (right) (55.9%). During the visual inspection of auricular signals, both ‘oily complexion’ (both ears) and ‘presence of ear hairs’ (left) had the highest specificity predictive values (86.3%). The ‘oily complexion’ of the ears also had the highest positive predictive values (>74.0%). Tenderness of ‘endocrine’ (left) achieved the highest negative predictive value (61.7%), followed by tenderness of ‘heart’ (right, 60.8%) and ‘endocrine’ (right, 59.6%) (Table 5).

## 4. Discussion

Dr. Paul Nogier, a French physician, has established the correspondences between various auricle parts and internal organs of the body, and he hypothesised that the auricle points were organised in the form of a homunculus similar to an inverted foetus [7]. This idea is in agreement with an ancient Chinese medical text ‘Divine Pivot’ (also called *‘Lingshu Jing’*), which stated that ‘the ears are the confluence of the channels’. This implies that the ears are related to the internal organs through the channels and collaterals, and they are directly or indirectly associated with the meridians that run over the body [8,20]. This case–control study has adopted a systematic and scientific approach using visual inspection, ESRT, and PPT to investigate auricular signals and their association with MS status. 

### 4.1. Visual Inspection

More participants in the MS+ group exhibited ‘oily complexion’ of the auricles compared with those in the MS− group. The pathway for the development of this auricular sign in MS+ cases is not clearly elucidated. It may be that people with MS have central obesity and higher triglyceride levels. From the TCM perspectives, these abnormalities are some kind of ‘dampness’ (fluid retention) accumulated in the body. If the body has excessive ‘dampness’, then excess moisture on the ears may appear [7]. Therefore, the oily complexion of the auricles might be a sign of ‘dampness’ owing to MS pathophysiology. 

Although no significant association was observed in the ear hairs and ear lobe creases between groups, these auricular signals were related to a number of MS risk factors, including smoking consumption, family history of having diabetes, central obesity, and various chronic illnesses (e.g., diabetes, hypertension, hyperlipidaemia, and CHD). It is worth noting that over 46% of MS− participants already have two or more of the abnormalities indicated in the IDF criteria, although they do not have central obesity. One reason for this finding is that MS− participants may possibly be at the pre-MS stage; hence, no significant group differences in the auricular signals were noted. The overall pre-MS prevalence was 21.9%, and this disease was more prevalent in men than in women and increased with age [21]. In a study conducted on 1,649 subjects who were asymptomatic but manifested known risk factors of diabetes, the odds ratio of acquiring diabetes increased from 3.7 in subjects with one risk factor to 28.4 in subjects with four or five risk factors [22]. Therefore, earlier screening during the pre-MS status deserves attention.

The relationship between ear lobe crease and premature cardiovascular disease was first described by Frank in 1973 [17]. Several positive claims that support the independent association of ear lobe crease with CHD prevalence and severity have been widely reported in recent years [23,24]. Suen et al. [11] found that participants with CHD have an ear lobe crease compared with those without CHD; moreover, these patients showed significantly higher conductivity and tenderness in the ‘heart’ point of both ears than the controls. In addition, the presence of ear hairs is a sign of hormonal changes that accompany the decline of kidney *Qi* (energy flow), which occurs with aging. Excessive hair growth in the meatus externa was also determined to be correlated to CHD [25]. 

### 4.2. Electrical Skin Resistance Measurement

Participants with MS displayed a significantly higher ‘endocrine’ (right) conductivity compared with the participants in the control group. In addition, the electrical conductivity of the acupoints under testing was significantly associated with the risk factors of MS.

The electrical resistance in the corresponding auricular points decreases when a disease or disorder is present in the body, and areas with lower electrical resistance than the reference points are considered to have either positively or highly conductive electrical resistance [10]. From a physiological perspective, the changes in the electrical resistance of the auricular points can be the result of the changes in the electrical resistance of the underlying cell membranes [26]. The electrical resistance on the cell membranes of a particular body system would be low when it is not functioning well. In a study conducted by Oleson [27] on patients with musculoskeletal pain, he hypothesised that a somatotopic organisation of the body is represented on the human auricle, and such alterations in skin conductivity at painful areas of the body have been attributed to the regional hyperactivity of the sympathetic nervous system. Moreover, sudomotor sympathetic nerve activation may change the skin’s moisture level and result in a decreased electrical resistance [28]. 

### 4.3. Tenderness Testing

The participants of the MS group experienced significant tenderness on the acupoints of ‘heart’, ‘pancreas and gallbladder’, and ‘endocrine’. Tenderness of ‘pancreas and gallbladder’ was associated with several risk factors of MS. Among the three examination methods adopted in the current study, tenderness on the specific acupoints has the highest sensitivity for MS status prediction.

The degree of tenderness is usually related to the severity of the condition; that is, the more sensitive the point, the more severe the disorder [8,29]. In an animal experiment, Oleson [30] found that the skin acupuncture points on dog bodies contain significantly higher substance P concentrations than the control skin points. Substance P is a spinal neurotransmitter that helps in pain transmission and triggers subcutaneous histamine release. Thus, an increase in substance P concentration would decrease the pain threshold, causing tenderness in the auricular points when touched [30].

### 4.4. Implications of the Findings

The ear is a valuable tool for revealing constitutional predispositions. The auricular diagnostic method, if deemed effective, is a simple, effective, and inexpensive complementary approach that could integrate Chinese and Western models of care in screening MS status. Auricular diagnosis is speculated to have a pre-diagnostic value and to possess a role in the secondary level of disease prevention.

This screening approach can be further tested in populations who have high risk of MS, such as those with family history of diabetes mellitus, hypertension, CHD, hyperlipidaemia, and central obesity, to perform secondary screening. If MS can be identified at an earlier stage using this simple, effective, and inexpensive complementary approach, appropriate treatment and lifestyle changes could be made earlier to prevent MS progression. 

### 4.5. Study Limitations

Although the association between certain auricular points and the risk factors of MS is suggested in this study, the mechanisms leading to the development and the time of onset of these signals remain uncertain. The issue on whether or not the difference between auricular signals appeared on one side or both sides is also unclear. A prospective cohort study should be conducted in the future to follow up vulnerable populations with MS risk to identify changes in the auricular signals that may have appeared during disease progression. This study was performed on a sample limited to the Chinese population. Therefore, further investigations must be performed with a larger sample to validate the use of auricular signals in Western populations or other ethnic groups.

## 5. Conclusions

The results of this study suggest that electrical conductivity and tenderness of a number of auricular points, including the ‘heart’, ‘pancreas and gall bladder’, and ‘endocrine’, are associated with MS and its risk factors. Tenderness on these auricular points has a relatively high sensitivity value. However, visual inspection of the ears to determine the presence of oily complexion and ear hairs has a higher specificity on MS risk prediction compared with the other approaches. Further investigations with a larger sample size could be conducted to verify the value of these auricular signals on MS risk prediction so that this method can be used as an early screening method for the population with a high MS risk.

## Figures and Tables

**Table 1:** The sociodemographic, family, and medical conditions of the participants.

Variables	MS+ (*n* = 102)	MS− (*n* = 102)	Test Statistics McNemar (unless indicated)
Age (mean 61.16, sd 10.33)	61.47 (10.109)	60.88 (10.551)	*p* > 0.05 (Paired *t*-test)
Gender 1 = male 2 = female	51 51	51 51	*p* > 0.05
Employment status 1 = full time 2 = part time 3 = retired/housewife/unemployed	37 16 49	44 13 45	*p* > 0.05
Smoking status 1 = never 2 = given up, occasionally, regularly	72 30	76 26	*p* > 0.05
Alcohol consumption No Social drinker Regular	66 31 5	61 33 8	*p* > 0.05 (Wilcoxon)
Body mass index (kg/m^2^)	27.06 (3.87)	22.85 (2.64)	*p* < 0.001 *** (paired *t*-test)
Central obesity (male) 0 = no 1 = yes	0 51	51 0	*p* < 0.001 ***
Central obesity (female) 0 = no 1 = yes	0 51	51 0	*p* < 0.001 ***
Waist circumference (cm)	95.58 (10.17)	82.86 (7.41)	*p* < 0.001 *** (paired *t*-test)
HDL-cholesterol (mmol/L)	1.24 (0.31)	1.47 (0.42)	*p* < 0.001 *** (paired *t*-test)
LDL-cholesterol (mmol/L)	2.84 (1.04)	2.92 (0.96)	*p* > 0.05 (paired *t*-test)
Triglycerides (mmol/L)	1.72 (1.04)	1.13 (0.64)	*p* < 0.001 *** (paired *t*-test)
Fasting glucose (mmol/L)	6.92 (2.24)	6.41 (2.52)	*p* > 0.05 (paired *t*-test)
Blood pressure (mmHg) SBP DBP	142.51 (17.97) 81.15 (11.45)	139.03 (21.43) 81.54 (12.67)	*p* > 0.05 *p* > 0.05 (paired *t*-test)
Family history on hyperlipidemia 1 = no 2 = yes	77 25	88 14	*p* < 0.05 *
Family history on diabetes 1 = no 2 = yes	54 48	73 29	*p* < 0.01 **
Family history on coronary heart disease 1 = no 2 = yes	85 17	83 19	*p* > 0.05
Family history on hypertension 1 = no 2 = yes	35 67	58 44	*p* < 0.01 **
Having hyperlipidemia 1 = no 2 = yes	31 71	68 34	*p* < 0.05 *
Having diabetes 1 = no 2 = yes	27 75	57 45	*p* < 0.01 **
Having coronary heart disease 1 = no 2 = yes	91 11	86 16	*p* > 0.05
Having hypertension 1 = no 2 = yes	40 62	63 39	*p* < 0.01 **
Comorbid illness 0 = no 1 = yes	10 92	30 72	*p* < 0.001***
Number of IDF criteria (cases, with central obesity) # Central obesity + 2 Central obesity + 3	47 55		
Number of IDF criteria (controls, with no central obesity) # 0 1 2 3 4	26 29 13 14 20		

* Statistically significant at 0.05; ** Statistically significant at 0.01; *** Statistically significant at 0.001; # IDF criteria: The International Diabetes Federation Criteria. Metabolic syndrome is defined as having central obesity plus any two of the following criteria: 1) wrist circumference: Asian ≥90 cm (men); ≥80 cm (women); 2) Raised triglycerides: >150 mg/dL (1.7 mmol/L), or specific treatment for this lipid abnormality; 3) Reduced high density lipoprotein cholesterol: <40 mg/dL (1.03 mmol/L) in males, <50 mg/dL (1.29 mmol/L) in females, or specific treatment for this lipid abnormality; 4) Raised blood pressure (BP): systolic blood pressure >130 mmHg or diastolic blood pressure >85 mmHg, or treatment of previously diagnosed hypertension; 5) If fasting plasma glucose >5.6 mmol/L (100 mg/dL), an oral glucose tolerance test is strongly recommended, but is not necessary to define the presence of the syndrome.

**Table 2:** Auricular signals between cases and controls.

Auricular Signals	MS+ (*n* = 102)	MS− (*n* = 102)	Test Statistics (McNemar Test)
**Visual inspection**			
Oily complexion of ears (Rt) No Yes	62 40	88 14	*p* < 0.001 ***
Oily complexion of ears (Lt) No Yes	61 41	88 14	*p* < 0.001 ***
Ear hairs (Rt) 1 = Absent 2 = Present	83 19	76 26	*p* > 0.05
Ear hairs (Lt) 1 = Absent 2 = Present	82 20	77 25	*p* > 0.05
Ear lobe crease (Rt) 0 = Absent 1 = Present	70 32	57 45	*p* > 0.05
Ear lobe crease (Lt) 0 = Absent 1 = Present	65 37	61 41	*p* > 0.05
**Electrical skin resistance test**			
Heart (Rt) 1 = Absent 2 = Present	56 46	52 50	*p* > 0.05
Heart (Lt)v1 = Absent 2 = Present	54 48	60 42	*p* > 0.05
Pancreas and gall bladder (Rt) 1 = Absent 2 = Present	81 21	84 18	*p* > 0.05
Pancreas and gall bladder (Lt) 1 = Absent 2 = Present	78 24	78 24	*p* = 1.000
Endocrine (Rt) 1 = Absent 2 = Present	56 46	71 31	*p* < 0.05 *
Endocrine (Lt) 1 = Absent 2 = Present	58 44	64 38	*p* > 0.05
**Pain pressure test**			
Heart (Rt) No Yes	40 62	60 42	*p* < 0.01 **
Heart (Lt) No Yes	41 61	70 32	*p* < 0.001 ***
Pancreas and gall bladder (Rt) No Yes	45 57	59 43	*p* > 0.05
Pancreas and gall bladder (Lt) No Yes	49 53	63 39	*p* > 0.05
Endocrine (Rt) No Yes	40 62	58 44	*p* < 0.05 *
Endocrine (Lt) No Yes	41 61	66 36	*p* < 0.01 ***

* Statistically significant at 0.05; ** Statistically significant at 0.01; *** Statistically significant at 0.001; Rt = right ear; Lt = left ear.

**Table 3:** Association of auricular signals and risk factors of metabolic syndrome # (*n* = 204).

	Age	Gender	Smoking	Alcohol Consumption	Family History (DM)	Diabetes	Hypertension	CHD	Hyperlipidemia	Central Obesity	Comorbid Illnesses
**Visual inspection**											
Oily complexion on ears (Rt)					*p* < 0.01			*p* < 0.05		*p* < 0.05	
Oily complexion on ears (Lt)					*p* < 0.05			*p* < 0.05		*p* < 0.001	
Ear hairs (Rt)		*p* < 0.001	*p* < 0.001		*p* < 0.01	*p* < 0.01			*p* < 0.05	*p* < 0.05	
Ear hairs (Lt)		*p* < 0.001	*p* < 0.001		*p* < 0.01	*p* < 0.001			*p* < 0.05	*p* < 0.05	
Ear lobe crease (Rt)	*p* < 0.001					*p* < 0.05			*p* < 0.01	*p* < 0.01	
Ear lobe crease (Lt)	*p* < 0.001					*p* < 0.05	*p* < 0.05	*p* < 0.05		*p* < 0.01	
**Electrical skin resistance test**											
Heart (Rt)	*p* < 0.05	*p* < 0.001	*p* < 0.01		*p* < 0.05	*p* < 0.001	*p* < 0.01	*p* < 0.05	*p* < 0.01	*p* < 0.05	*p* < 0.001
Heart (Lt)		*p* < 0.001				*p* < 0.001	*p* < 0.01	*p* < 0.05	*p* < 0.01		*p* < 0.001
Pancreas and gallbladder (Rt)			*p* < 0.05		*p* < 0.05	*p* < 0.01					
Pancreas and gallbladder (Lt)		*p* < 0.01	*p* < 0.01		*p* < 0.05	*p* < 0.001	*p* < 0.01	*p* < 0.05	*p* < 0.01		*p* < 0.01
Endocrine (Rt)		*p* < 0.001	*p* < 0.01			*p* < 0.001	*p* < 0.001		*p* < 0.01		*p* < 0.001
Endocrine (Lt)	*p* < 0.05	*p* < 0.001	*p* < 0.001		*p* < 0.05	*p* < 0.001	*p* < 0.001	*p* < 0.05	*p* < 0.01		*p* < 0.001
**Pain pressure test**											
Heart (Rt)				*p* < 0.05		*p* < 0.001					
Heart (Lt)											
Pancreas and gallbladder (Rt)						*p* < 0.001	*p* < 0.05		*p* < 0.05		*p* < 0.05
Pancreas and gallbladder (Lt)		*p* < 0.01								*p* < 0.05	
Endocrine (Rt)										*p* < 0.05	*p* < 0.05
Endocrine (Lt)						*p* < 0.05			*p* < 0.05	*p* < 0.01	

**#** Using chi-square analyses; Only statistical significant p-values were displayed; Rt = right ear; Lt = left ear; DM= diabetes mellitus; CHD = Coronary heart disease.

**Table 4:** Association between auricular signals under testing # (*n* = 204).

Visual inspection (V)	Va	Vb	Vc	Vd	Ve	Vf	Ea	Eb	Ec	Ed	Ee	Ef	Pa	Pb	Pc	Pd	Pe	Pf
Va	---	***												*				
Vb		---												*				
Vc			---	***			**			*		***				**		
Vd			***	---		*	***		*	**		***				*		
Ve					---	***	**					*						**
Vf				*	***	---	*		*			*						*
**Electrical skin resistance test (E)**																		
Ea			**	***	**	*	---	***	***	***	***	***						***
Eb							***	---	***	***	***	***						
Ec				*		*	***	***	---	***	***	***			*			*
Ed			*	**			***	***	***	---	***	***						
Ee							***	***	***	***	---	***						
Ef			***	***	*	*	***	***	***	***	***	---						
**Pain pressure test (P)**																		
Pa													---	*	***	**	***	***
Pb	*	*											*	---				
Pc									*				***		---	***	***	**
Pd			**	*									**		***	---	***	***
Pe													***		***	***	---	*
Pf					**	*	***		*				***		**	***	*	---

# Using chi-square analyses; Only statistical significant *p*-values were displayed (* *p* < 0.05; ** *p* < 0.01, *** *p* < 0.001). Visual inspection (V): Va = oily complexion on ears (right); Vb = oily complexion on ears (left); Vc = ear hairs (right); Vd = ear hairs (left); Ve = ear lobe crease (right); Vf = ear lobe crease (left). Electrical skin resistance test (E): Ea = heart (right); Eb = heart (left); Ec = pancreas and gallbladder (right); Ed = pancreas and gallbladder (left); Ee = endocrine (right); Ef = endocrine (left). Pain pressure test (P): Pa = heart (right); Pb = heart (left); Pc = pancreas and gallbladder (right); Pd = Pancreas and gallbladder (left); Pe = endocrine (right); Pf = endocrine (left).

**Table 5:** Predictive power of auricular signals on the risk of metabolic syndrome (*n* = 204).

Auricular Signals	Sensitivity (%)	Specificity (%)	Positive Predictive Value (%)	Negative Predictive Value (%)
**Visual inspection**				
Oily complexion on ears (Rt)	39.2	**86.3**	**74.1**	58.7
Oily complexion on ears on ears (Lt)	40.2	**86.3**	**74.5**	59.1
Ear hairs (Rt)	14.7	**77.5**	39.5	47.6
Ear hairs (Lt)	15.7	**86.3**	39.0	47.2
Ear lobe crease (Rt)	30.4	53.9	39.7	43.7
Ear lobe crease (Lt)	36.3	58.8	46.8	48.0
**Electrical skin resistance test**				
Heart (Rt)	46.0	51.0	48.5	48.6
Heart (Lt)	47.1	59.8	53.9	53.0
Pancreas and gallbladder (Rt)	20.6	**82.4**	53.8	50.9
Pancreas and gallbladder (Lt)	24.5	76.5	51.0	50.3
Endocrine (Rt)	46.1	69.6	60.3	56.3
Endocrine (Lt)	43.1	62.7	53.7	52.5
**Pain pressure test**				
Heart (Rt)	**60.8**	60.8	61.4	**60.8**
Heart (Lt)	31.4	70.6	52.5	50.7
Pancreas and gallbladder (Rt)	**55.9**	60.8	59.4	57.9
Pancreas and gallbladder (Lt)	52.0	62.7	58.9	56.6
Endocrine (Rt)	**60.8**	57.8	59.6	**59.6**
Endocrine (Lt)	**59.8**	64.7	**63.5**	**61.7**

Sensitivity (true positive) = number of true positives/(number of true positives + number of false negatives). Specificity (true negative) = number of true negatives/(number of true negatives + number of false positives). Positive predictive value = number of true positives/(number of true positives + number of false positives). Negative predictive value = number of true negatives/(number of true negatives + number of false negatives). The top 3 values in each test are shown in bold.
